# Bevacizumab in combination with chemotherapy for the treatment of advanced ovarian cancer: a systematic review

**DOI:** 10.1186/1757-2215-7-57

**Published:** 2014-05-19

**Authors:** Gerasimos Aravantinos, Dimitrios Pectasides

**Affiliations:** 1 Second Department of Medical Oncology, Agioi Anargiroi Cancer Hospital, Κifisia, Athens, Greece; 2 Second Department of Internal Medicine, Hippokration Hospital, University of Athens School of Medicine, Athens, Greece

**Keywords:** Angiogenesis, Bevacizumab, Fallopian tube cancer, Ovarian cancer, Primary peritoneal cancer, Targeted therapies

## Abstract

As increased angiogenesis has been linked with the progression of ovarian cancer, a number of anti-angiogenic agents have been investigated, or are currently in development, as potential treatment options for patients with advanced disease. Bevacizumab, a recombinant monoclonal antibody against vascular endothelial growth factor, has gained European Medicines Agency approval for the front-line treatment of advanced epithelial ovarian cancer, fallopian tube cancer or primary peritoneal cancer in combination with carboplatin and paclitaxel, and for the treatment of first recurrence of platinum-sensitive ovarian cancer in combination with carboplatin and gemcitabine. We conducted a systematic literature review to identify available efficacy and safety data for bevacizumab in ovarian cancer as well as for newer anti-angiogenic agents in development. We analyzed published data from randomized, controlled phase II/III clinical trials enrolling women with ovarian cancer to receive treatment with bevacizumab. We also reviewed available data for emerging anti-angiogenic agents currently in phase II/III development, including trebananib, aflibercept, nintedanib, cediranib, imatinib, pazopanib, sorafenib and sunitinib. Significant efficacy gains were achieved with the addition of bevacizumab to standard chemotherapy in four randomized, double-blind, phase III trials, both as front-line treatment (GOG-0218 and ICON7) and in patients with recurrent disease (OCEANS and AURELIA). The type and frequency of bevacizumab-related adverse events was as expected in these studies based on published data. Promising efficacy data have been published for a number of emerging anti-angiogenic agents in phase III development for advanced ovarian cancer. Further research is needed to identify predictive or prognostic markers of response to bevacizumab in order to optimize patient selection and treatment benefit. Data from phase III trials of newer anti-angiogenic agents in ovarian cancer are awaited.

## Introduction

Ovarian cancer is the seventh most common cancer in women [1], with an estimated 225,500 new cases and 140,200 deaths globally in 2008 [2]. Symptoms of the disease are non-specific, including abdominal discomfort or fullness, dyspepsia, and bloating, which may mimic other conditions and lead to a delay in diagnosis [1]. Consequently, 75% of women are diagnosed with advanced disease (International Federation of Gynecology and Obstetrics [FIGO] stage III or IV) [3], which has a median overall survival (OS) of 15–23 months and an estimated 5-year survival of just 20% [4]. Around 90% of all ovarian cancers are epithelial ovarian cancers (EOCs) and are believed to arise from the ovarian surface epithelium or mullerian derivatives, including the fallopian tube; primary ovarian cancers also include ovarian-type peritoneal tumors [4].

### Current treatment options

Surgery is effective in most cases of early stage ovarian cancer (FIGO stage I-IIA) with a 5-year survival rate of around 90% [5]. Adjuvant chemotherapy for well-staged early stage ovarian cancer is controversial [4] but some studies have shown a benefit [6]. Clinical practice guidelines developed by ESMO recommend six cycles of single-agent carboplatin as adjuvant treatment in patients with intermediate and high-risk early stage ovarian cancer [4].

After surgical cytoreduction, the treatment of choice for patients with advanced EOC (FIGO stage IIB-IIIC) is platinum-based chemotherapy (six cycles of carboplatin plus paclitaxel [CP] given every 3 weeks) [4]. Recently a modified CP regimen with weekly paclitaxel resulted in better long-term outcome than the 3-weekly regimen in a phase III study in Japanese women with advanced ovarian cancer [7,8], with confirmatory findings reported in European women in the randomized, multicenter phase III MITO-7 study [9], and in the chemotherapy arm of the phase III GOG-0262 trial [10]. This regimen has now been included in the NCCN treatment guidelines [11]. Although approximately 80% of patients respond to front-line chemotherapy, more than 70% of patients with advanced stage disease recur within 5 years and develop drug resistance [12,13].

For recurrent disease, the treatment choice is based on the timing and nature of the recurrence and the extent of prior chemotherapy [4]. Platinum-sensitive patients with a treatment-free interval > 24 months and good performance status should be considered for surgical resection [4]. Patients responding to front-line platinum-containing chemotherapy are very likely to respond to a rechallenge with platinum-based therapies. However, patients relapsing after front-line platinum-paclitaxel chemotherapy are at risk of significant neurotoxicity if retreated within 12 months with the same regimen. Rechallenge with platinum-based therapies in patients with platinum-refractory disease yields a response rate of only 10%. Similarly low response rates have also been reported with a number of agents in platinum-paclitaxel-refractory disease [4]. More effective treatment strategies, particularly molecular targeted agents, are required to improve outcomes for women with advanced ovarian cancer.

### Angiogenesis and ovarian cancer

The vascular endothelial growth factor (VEGF) family consists of VEGF-A (often referred to as VEGF), VEGF-B, VEGF-C, VEGF-D, placental growth factor (PlGF), VEGF-E and VEGF-F [14]. VEGF-mediated angiogenesis plays a vital role in normal ovarian function, controlling the cyclical growth of ovarian follicles and the development and maintenance of the corpus luteum [14,15]. However, there is a well-established association between VEGF overexpression, increased angiogenesis and the development and progression of ovarian cancer. In a literature review of nine studies including 529 patients with newly diagnosed ovarian cancer, high serum VEGF levels correlated with higher risk of death or recurrence [16]. Five of the studies found serum VEGF to be an independent prognostic factor for OS in multivariate analyses. In at least one of the studies, a statistically significant association was identified between serum VEGF and FIGO stage, tumor grade and size, lymph node involvement and presence of ascites. VEGF has also been implicated in the peritoneal dissemination of ovarian cancer and the development of malignant ascites [17] which is inversely linked with survival [14,18].

Given the association between increased angiogenesis and the progression of ovarian cancer, a number of anti-angiogenic agents are currently in development as potential treatment options for patients with advanced disease. Bevacizumab is a recombinant, humanized, monoclonal antibody that binds to all isoforms of VEGF [19]. Bevacizumab is indicated for the treatment of several solid tumors in combination with cytotoxic chemotherapy, including non-small cell lung cancer, metastatic colorectal cancer, metastatic renal cell carcinoma, metastatic breast cancer (EU only) and glioblastoma. Bevacizumab has gained European Medicines Agency approval, in combination with CP, for the front-line treatment of patients with advanced EOC, fallopian tube cancer (FTC) or primary peritoneal cancer (PPC), and, in combination with carboplatin and gemcitabine (CG), for the treatment of first recurrence of platinum-sensitive ovarian cancer.

The purpose of this systematic review was to summarize available efficacy and safety data for bevacizumab in ovarian cancer and to highlight data for emerging anti-angiogenic agents in phase II/III development.

#### Search strategy

We designed a systematic literature review to identify published randomized, controlled, prospective phase II/III clinical trials of bevacizumab in women aged ≥ 18 years with histologically proven EOC, FTC or PPC and no concurrent malignancies. We also searched for studies of promising new anti-angiogenic agents in ovarian cancer.

PubMed/Medline and Embase databases were searched from 1 January 2002 to 8 November 2013, using the terms: AEE788; aflibercept; AMG 386; angiogenesis inhibitors; anti-VEGF; bevacizumab; BIBF 1120; cediranib; imatinib; nintedanib; pazopanib; perifosine; sorafenib; sunitinib; trebananib; vascular endothelial growth factor; VEGF-receptor AND ovarian cancer OR fallopian tube cancer OR primary peritoneal cancer. Congress abstracts from ASCO, ECCO-ESMO and SGO were also searched for these agents from 1 January 2009 to 8 November 2013. Results were limited to peer-reviewed, English language articles only. Reviews, meta-analyses, case reports, editorials, and letters were excluded.

From a total of 176 articles and 204 abstracts identified in the search, 57 articles and 98 abstracts met the criteria for inclusion.

### Bevacizumab in advanced ovarian cancer

#### Phase II trial data

One randomized phase II trial of bevacizumab was identified. The STAT study is examining the efficacy of front-line bevacizumab plus erlotinib consolidation therapy following CP plus bevacizumab induction therapy in patients with EOC, FTC or PPC [20]. Of 60 enrolled patients, 12 were taken off study prior to randomization, leaving 23 patients in the bevacizumab group and 25 patients in the bevacizumab plus erlotinib group. Overall, 6 patients achieved a complete response, 26 had a partial response, and 20 had stable disease. Progression-free survival (PFS) data from the study are not yet mature.

#### Phase III trial data

Efficacy data are available from four randomized, double-blind, phase III trials of bevacizumab in advanced ovarian cancer: GOG-0218 [21] and ICON7 [22,23] in the front-line treatment setting and OCEANS [24,25] and AURELIA [26,27] in patients with recurrent disease (Table 1).

**Table 1:** Summary of efficacy data from randomized, controlled phase III trials of bevacizumab in advanced ovarian cancer

**Study (n)**	**Regimen**	**ORR (CR + PR), %**	**Median PFS, months**	**Median OS, months**
		**[p value]**	**[HR; p value]**	**[HR; p value]**
GOG-0218 [21]	CP + placebo vs. CP + Bev vs. CP + Bev → Bev maintenance	–	10.3 vs. 11.2 vs. 14.1	39.3 vs. 38.7 vs. 39.7
(n = 1,873)			[0.908; 0.16]^a^	[1.036; 0.76]^a^
			[0.717; < 0.001]^b^	[0.915; 0.45]^b^
ICON7 [22,23]	CP vs. CP + Bev → Bev maintenance	48 vs. 67	17.4 vs. 19.8	Restricted mean survival time, months
(n = 1,528)		[< 0.001]	[0.87; 0.04]	44.6 vs. 44.5
OCEANS [24,25]	CG + placebo vs. CG + Bev	57.4 vs. 78.5	8.4 vs. 12.4	33.7^c^ vs. 33.4^c^
(n = 484)		[< 0.0001]	[0.484; < 0.0001]	[0.960; 0.736]
AURELIA [26,27]	CTx (PLD, P or Top) vs. CTx + Bev	12.6 vs. 30.9	3.4 vs. 6.7	13.3 vs. 16.6
(n = 361)		[0.001]	[0.48; < 0.001]	[0.85; 0.174]

Bev = bevacizumab. C = carboplatin. CR = complete response. CTx = chemotherapy. G = gemcitabine. HR = hazard ratio. ORR = overall response rate. OS = overall survival. P = paclitaxel. PFS = progression-free survival. PLD = pegylated liposomal doxorubicin. PR = partial response. Top = topotecan.

^a^CP + Bev vs. CP + placebo.

^b^CP + Bev → Bev vs. CP + placebo.

^c^Interim data.

#### GOG-0218

**Figure 1:**
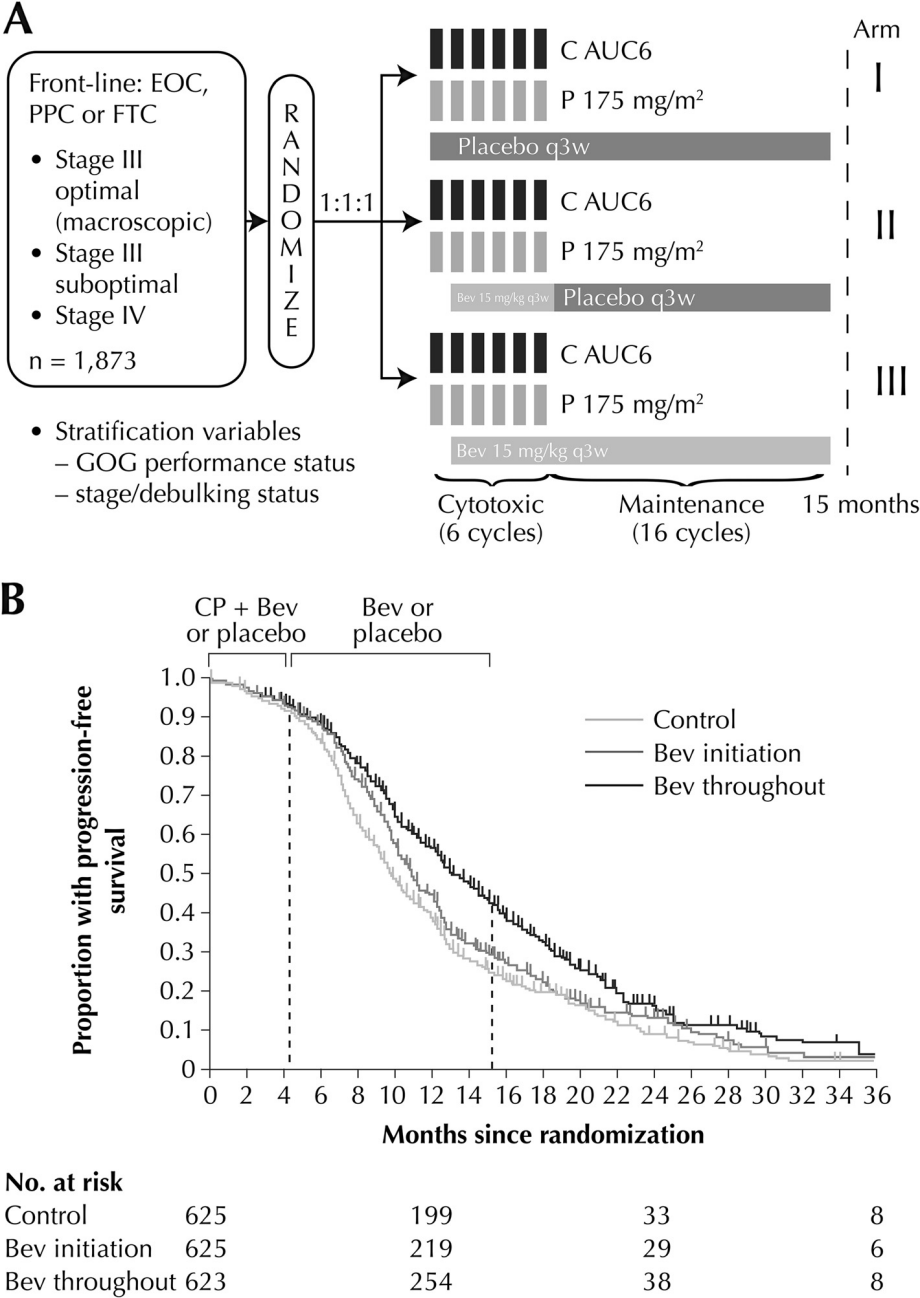
**GOG-0218 randomized, double-blind, placebo-controlled phase III trial: (A) study design; (B) progression-free survival analysis (reproduced with permission) **[21]**.** EOC = epithelial ovarian cancer. PPC = primary peritoneal cancer. FTC = fallopian tube cancer. C = Carboplatin. AUC = area under the curve. P = Paclitaxel. GOG = Gynecologic Oncology Group. Bev = bevacizumab. q3w = once every 3 weeks.

The GOG-0218 study enrolled 1,873 women with newly diagnosed stage III (incompletely resectable) or stage IV EOC to receive: CP with placebo (*n* = 625); CP with bevacizumab from cycles 2–6 and placebo from cycles 7–22 (bevacizumab initiation, *n* = 625); or CP with bevacizumab (bevacizumab throughout, *n* = 623) (Figure 1A). At the time of the primary analysis, 76.3% of patients were alive with a median follow-up of 17.4 months [21]. Compared with the control arm, the primary endpoint of PFS was longer in the bevacizumab initiation arm (hazard ratio [HR] 0.908; *p* = 0.16; median 11.2 vs. 10.3 months) and significantly longer in the bevacizumab throughout arm (HR 0.717; *p* < 0.001; median 14.1 vs. 10.3 months) (Table 1). The maximum separation of the survival curves for the control group and the bevacizumab throughout group occurred at 15 months, with convergence around 9 months later (Figure 1B). A pre-specified CA-125 censored analysis (progression events based only on CA-125 criteria were not counted as events) showed a median PFS of 12.0 months in the control group and 18.0 months in the bevacizumab throughout group (HR 0.645; *p* < 0.001) [21]. An Independent Review Committee (IRC) confirmed these findings, reporting a median PFS of 13.1 months in the control arm versus 19.1 months in the bevacizumab throughout arm (HR 0.630; *p* < 0.0001) [28]. A consistent treatment effect was observed across patient subgroups stratified by age, performance status, tumor grade, histologic type and cancer stage [21]. OS, however, was not significantly different between the bevacizumab-containing treatment arms and the control arm (Table 1). Quality of life (QoL), assessed in 1,693 eligible patients using the Trial Outcome Index (TOI) of the Functional Assessment of Cancer Therapy-Ovary (FACT-O) questionnaire, improved from baseline to cycle 13 in all treatment groups. Patients receiving bevacizumab had lower mean FACT-O TOI scores during chemotherapy than patients in the control group (2.7 points and 3.0 points lower for the initiation and throughout groups, respectively; *p* < 0.001 in both cases), but no significant between-group differences were observed after the completion of chemotherapy [21].

#### ICON7

**Figure 2:**
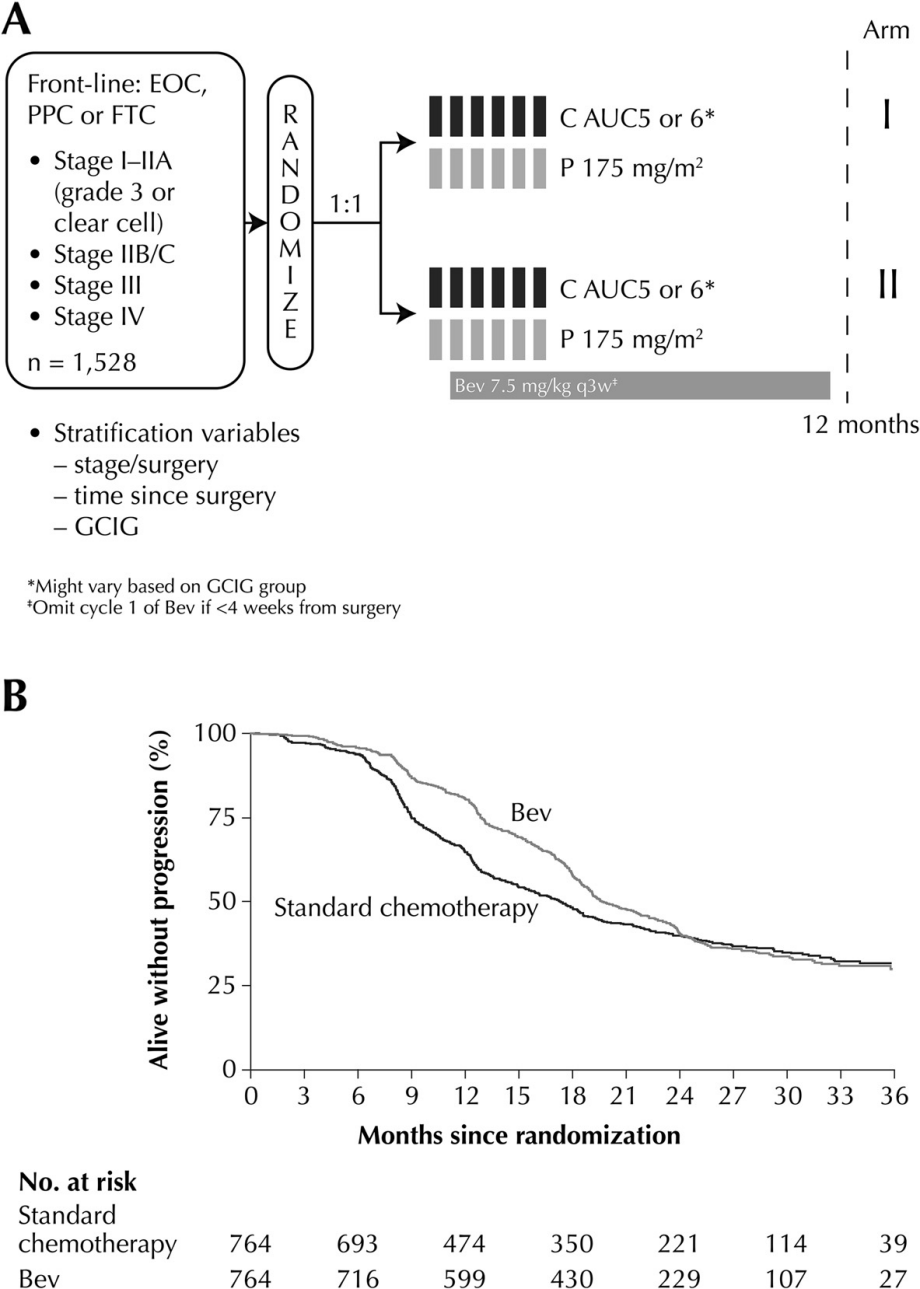
**ICON7 randomized, double-blind, placebo-controlled phase III trial: (A) study design (B) Updated progression-free survival analysis (reproduced with permission) [**[22]**].** EOC = epithelial ovarian cancer. PPC = primary peritoneal cancer. FTC = fallopian tube cancer. C = Carboplatin. AUC = area under the curve. P = Paclitaxel. Bev = bevacizumab. CGIG = Gynecologic Cancer InterGroup. q3w = once every 3 weeks.

In the ICON7 study, 1,528 women with high-risk early stage ovarian cancer (9% of the study population) or advanced EOC, FTC or PPC were randomized to receive front-line CP (*n* = 753) or CP plus bevacizumab followed by bevacizumab for a maximum of 12 months (*n* = 745) (Figure 2A). Median follow-up was 19.4 months with 759 progression or death events. A statistically significant increase in PFS, the primary endpoint, was noted in the bevacizumab arm relative to the CP arm (HR 0.81, 95% confidence interval [CI]: 0.70–0.94; *p* = 0.004; median 19.0 vs. 17.3 months) [22]. Similar results were obtained in an updated analysis of PFS and OS data after a median follow-up of 28 months with 934 progression or death events reported (Table 1). The effect of bevacizumab changed over time, with maximal benefit at 12 months, which diminished by 24 months post-randomization (Figure 2B). A significantly higher objective response rate (ORR) was observed with bevacizumab plus CP versus CP alone (*p* < 0.001) (Table 1). Final OS results from the trial, after a median follow-up of 49 months, were reported as a restricted mean survival time improvement of 0.9 months from 44.6 months with CP alone to 45.5 months with bevacizumab plus CP (*p =* 0.85 log-rank) [23]. QoL scores assessed by the European Organisation for Research and Treatment of Cancer QLQ-C30 and QLQ-OV28 questionnaires suggested clinically small, but statistically significant deficits in global QoL (*p* < 0.0001) following the addition of bevacizumab to CP [29].

#### OCEANS

The OCEANS study is assessing the efficacy and safety of CG with or without bevacizumab in patients with platinum-sensitive recurrent EOC, FTC or PPC. A total of 484 patients whose disease had recurred ≥ 6 months after front-line platinum-containing chemotherapy were randomized 1:1 to receive CG plus bevacizumab or CG plus placebo. At the time of the final PFS analysis, median follow-up was 24 months, with 338 events [24]. The addition of bevacizumab to CG significantly increased PFS, the primary endpoint, compared with placebo (*p* < 0.0001) (Table 1). These findings were confirmed by IRC assessment, which reported an increase in median PFS from 8.6 months to 12.3 months with the addition of bevacizumab (HR 0.451, 95% CI: 0.351–0.580; *p* < 0.0001) [24]. A statistically significant improvement in ORR was also observed in the bevacizumab group relative to placebo (Table 1), with a majority of partial responses reported (61.2% bevacizumab vs*.* 48.3% placebo). The duration of response was also longer in patients receiving bevacizumab than in those receiving placebo (10.4 vs*.* 7.4 months, respectively; HR 0.534, 95% CI: 0.408–0.698). No difference in OS was observed between the treatment groups at the third interim analysis of the data after a median follow-up of 42 months (Table 1) [25].

#### AURELIA

The AURELIA trial is investigating the combination of bevacizumab and chemotherapy in platinum-resistant recurrent ovarian cancer. A total of 361 patients with ovarian cancer whose disease had progressed ≤ 6 months after ≥ 4 cycles of platinum-based chemotherapy were randomized to receive chemotherapy alone or in combination with bevacizumab (pegylated liposomal doxorubicin [PLD], *n* = 126; topotecan, *n* = 120; or paclitaxel, *n* = 115) [26]. Median follow-up after 301 PFS events was 13.5 months [26]. A statistically significant and clinically meaningful improvement in PFS (*p* < 0.001), the primary study endpoint, and in ORR (*p* = 0.001) was observed in the bevacizumab plus chemotherapy group compared with the chemotherapy alone group (Table 1). No difference in OS was observed between the treatment groups at the final data analysis [27]. More patients receiving bevacizumab plus chemotherapy showed a ≥ 15% improvement in the QLQ-OV28 abdominal/gastrointestinal [GI] symptom subscale at Week 8/9 compared with those receiving chemotherapy alone (21.9% vs. 9.3%, respectively, 95% CI: 4.4–20.9; *p* = 0.002) [30].

Bevacizumab is being evaluated in a number of ongoing, randomized, phase III trials in ovarian cancer. The GOG-0241 study is comparing front-line CP with or without bevacizumab versus oxaliplatin and capecitabine with or without bevacizumab in patients with mucinous EOC or FTC [31]. GOG-0213 is enrolling patients with platinum-sensitive recurrent EOC, PPC or FTC who have undergone cytoreductive surgery to receive CP with or without bevacizumab, followed by bevacizumab and secondary cytoreductive surgery [32]. The AGO-OVAR 17 trial is evaluating the optimal treatment duration of front-line bevacizumab in combination with CP (15 vs. 30 months) in EOC, FTC or PPC [33]. GOG-0252 is examining bevacizumab combined with intravenous or intraperitoneal chemotherapy in stage II–IV ovarian cancer [34]. Finally, GOG-0262 is comparing the standard once every 3 weeks CP with dose-dense weekly paclitaxel in combination with carboplatin (Katsumata regimen) with or without concurrent and consolidation bevacizumab in EOC, PPC or FTC [35]. Prior to enrolment in GOG-0262, each patient decides whether their treatment will include concurrent and maintenance bevacizumab based on discussion of the risk to benefit ratio with their clinician. Safety results from the phase II OCTAVIA study examining front-line bevacizumab plus weekly paclitaxel plus once every 3 weeks carboplatin, followed by single-agent bevacizumab, are promising [36].

#### Biomarker analyses

The potential prognostic and predictive value of VEGF-A has been examined in studies of bevacizumab across multiple tumor types. In biomarker analyses of post-surgery samples from the GOG-0218 study, a lack of correlation was found between plasma VEGF-A levels and time since surgery [37]. However, in an exploratory analysis of a phase II study of bevacizumab and erlotinib in platinum-resistant ovarian cancer, a prognostic association between tumor VEGF-A expression and disease progression (PD) was identified (*p* = 0.03); for every 100-unit increase in the VEGF-A score, there was a 3.7-fold increase in the odds of progression (95% CI: 1.1–16.6) [38]. High baseline serum VEGF levels correlated with poor OS (*p* = 0.01) in a phase II study of bevacizumab in patients with chemotherapy-resistant ovarian cancer [39]. High serum levels of platelet-derived growth factor (PDGF)-BB and fibroblast growth factor 2 were of prognostic significance in this study, but none of the markers predicted response to bevacizumab. Further research is needed to clarify the role of clinical and biologic factors in predicting response to bevacizumab. Prognostic, but not predictive, biomarker data will be collected in the MANGO-2 phase IV trial [40] in patients with advanced ovarian cancer receiving front-line bevacizumab in combination with CP.

#### Safety of bevacizumab in advanced ovarian cancer

An overview of grade ≥ 3 adverse events (AEs) occurring in the four phase III trials of bevacizumab identified in the literature search is presented in Table 2. The type and frequency of bevacizumab-related AEs was as expected, with hypertension, thromboembolic events, proteinuria, bleeding and GI events occurring at a higher incidence in the bevacizumab-containing arms than in the control arms.

**Table 2:** Grade ≥ 3 adverse events occurring in randomized phase III trials of bevacizumab in advanced ovarian cancer

**Grade ≥ 3 AE, %**	***GOG-0218 *** [21]			***ICON7 *** [22]		***OCEANS *** [41]		***AURELIA *** [26]	
	**CP + placebo**	**CP + Bev**	**CP + Bev → Bev**	**CP**	**CP + Bev → Bev**	**CG + placebo**	**CG + Bev**	**CTx**	**CTx + Bev**
	**(*n*** **= 601)**	**(*n*** **= 607)**	**(*n*** **= 608)**	**(*n*** **= 753)**	**(*n*** **= 745)**	**(*n*** **= 233)**	**(*n*** **= 247)**	**(*n*** **= 182)**	**(*n*** **= 179)**
**Neutropenia**	57.7^a^	63.3^a^	63.3^a^	15	17	–	–	–	–
**Pain**	41.6^b^	41.5^b^	47.0^b^	–	–	–	–	–	–
**Thrombocytopenia**	–	–	–	2	3	34	40	–	–
**Hypertension**	7.2^b^	16.5^b^	22.9^b^	<1	6	0.4	17.8	–	–
**VTE**	5.8^c^	5.3^c^	6.7^c^	2	4	–	–	4	3
**Febrile neutropenia**	3.5^c^	4.9^c^	4.3^c^	2	3	–	–	1	1
**Proteinuria**	0.7	0.7	1.6	<1	1	0.9	9.7	–	–
**Bleeding (non-CNS)**	0.8	1.3	2.1	<1	1	0.9	5.7	1	1
**Wound healing complications**	2.8^c^	3.6^c^	3.0^c^	<1	1	–	–	–	–
**ATE**	0.8^c^	0.7^c^	0.7^c^	1	3	–	–	0	2
**GI events**	1.2^b^	2.8^b^	2.6^b^	<1	1	0	0	–	–
**Epistaxis**	–	–	–	–	–	0.4	4.9	–	–
**Abscess/fistula**	–	–	–	1	1	0.4^c^	1.6^c^	–	–
**RPLS**	0	0.2^c^	0.2^c^	0	0	0	0.8^c^	0	1
**CHF**	–	–	–	<1	<1	–	–	1	1
**CNS bleeding**	0	0	0.3^c^	0	<1	–	–	–	–

AE = adverse event. ATE = arterial thromboembolic event. Bev = bevacizumab. C = carboplatin. CHF = congestive heart failure. CNS = central nervous system. CTx = chemotherapy. G = gemcitabine. GI = gastrointestinal. P = paclitaxel. RPLS = reverse posterior leukoencephalopathy syndrome. VTE = venous thromboembolic event.

^a^Grade ≥ 4.

^b^Grade ≥ 2.

^c^All grades.

In the GOG-0218 study, grade ≥ 2 hypertension was significantly more common in patients receiving bevacizumab versus the control group (*p* < 0.001) but there were no significant differences in the incidence of the other treatment-emergent AEs. Fatal AEs were reported in 1.0%, 1.6% and 2.3% of patients in the control group, bevacizumab initiation group and bevacizumab maintenance group, respectively [21]. Grade ≥ 2 hypertension was reported more frequently in the bevacizumab-containing arm of the ICON7 study compared with the chemotherapy-alone arm (18% vs. 2%, respectively). Five deaths related to treatment or PD were reported on the ICON7 study: one in the chemotherapy arm (0.1%) and four in the bevacizumab arm (0.5%) [22].

Both the AURELIA and OCEANS trials have strict inclusion criteria designed to minimize the risk of GI perforations, based on prior observations that GI perforation risk was increased in patients with ovarian cancer recurrent after multiple lines of therapy. An updated safety analysis of the OCEANS study confirmed the findings at the time of the primary PFS analysis [41]. Higher incidences of grade ≥3 proteinuria and hypertension reported in the bevacizumab arm relative to the placebo arm (Table 2) were thought to be due to the longer treatment duration of bevacizumab. Bevacizumab-related GI perforations were minimized in the AURELIA study due to the strict inclusion criteria [26].

### Emerging anti-angiogenic agents in ovarian cancer

A number of investigational anti-angiogenic agents are currently in phase II/III development for the treatment of recurrent ovarian cancer. Table 3 summarizes available phase II/III efficacy data for these agents.

**Table 3:** Summary of efficacy data from phase II/III trials of emerging anti-angiogenic agents in recurrent ovarian cancer

**Study (n)**	**Regimen**	**ORR (CR + PR), %**	**Median PFS, months**	**Median OS, months**
Matulonis et al. [42]	Cediranib 45 mg/day	17	5.2	Not reached
(n = 46)				
Ledermann et al. [43]	Cediranib 20 mg/day → cedarinib maintenance vs. placebo	–	Restricted mean survival time, months	Restricted mean survival time, months
(n = 456)			11.4 vs. 9.4	20.3 vs. 17.6
Du Bois et al. [44]	Pazopanib 800 mg/day vs. placebo	–	17.9 vs. 12.3	Not reached
(n = 940)				
Campos et al. [45]	Sunitinib 37.5 mg/day	8.3	9.9^b^	–
(n = 35)				
Biagi et al. [46]	Sunitinib 50 mg/day (int.)	13.3	4.1	–
(n = 30)				
Baumann et al. [47]	Sunitinib 50 mg/day (int.) vs. 37.5 mg/day (cont.)	16.7 vs. 5.4	4.8 vs. 2.9	13.6 vs. 13.7
(n = 73)				
Matei et al. [48]	Sorafenib 400 mg b.i.d.	3.4	6-month PFS rate: 24%	–
(n = 71)				
Herzog et al. [49]	Sorafenib 400 mg b.i.d. vs. placebo	–	386 vs. 478^a^	–
(n = 249)				
Ledermann et al. [50]	Nintedanib 250 mg b.i.d. vs. placebo	–	36-week PFS rate:	–
(n = 83)			16.3% vs. 5.0%	
Du Bois et al. [51]	Nintedanib 200 mg b.i.d. + CP vs. nintedanib + placebo	–	17.3 vs. 16.6	–
(n = 1,366)				
Coleman et al. [52]	Aflibercept 6 mg/kg + D	54	6.2	24.3
(n = 46)				
Gotlieb et al. [53]	Aflibercept 4 mg/kg vs. placebo	–	6.3 vs. 7.3^b^	12.9 vs. 16.0^b^
(n = 55)				
Colombo et al. [54]	Aflibercept 4 mg/kg	–	8.5^b^	–
(n = 16)				
Karlan et al. [55]	Trebananib 10 mg/kg + P vs. trebananib 3 mg/kg + P vs. placebo + P	37 vs. 19 vs. 27	7.2 vs. 5.7 vs. 4.6	22.5 vs. 20.4 vs. 20.9
(n = 161)				
Monk et al. [56]	Trebananib 15 mg/kg + P vs. placebo + P	38 vs. 30	7.2 vs. 5.4	19.0^c^ vs. 17.3^c^
(n = 919)				

b.i.d. = twice daily. cont = continuous. CR = complete response. D = docetaxel. int = intermittent. ORR = objective response rate. OS = overall survival. PFS = progression-free survival. P = paclitaxel. PR = partial response.

^a^Time in days.

^b^Time in weeks.

^C^Interim data.

#### Trebananib

Trebananib (AMG 386) is an anti-angiopoietin peptide that blocks the interaction of angiopoietin-1 and -2 with the Tie2 receptor [55]. In a randomized, double-blind, phase II trial, 161 patients with recurrent ovarian cancer received weekly paclitaxel and were randomized 1:1:1 to receive trebananib (10 mg/kg or 3 mg/kg) or placebo until PD, unacceptable toxicity or withdrawal of consent. The combination regimen showed evidence of anti-tumor activity with greatest efficacy at the 10 mg/kg dose (Table 3) [55]. Grade ≥ 3 AEs occurring more frequently in the trebananib-containing arms than in the placebo arm were hypokalemia, peripheral neuropathy and dyspnea. A number of randomized, double-blind, phase III trials of trebananib are currently ongoing. TRINOVA-1 is investigating the combination of paclitaxel plus trebananib or placebo in recurrent partially platinum-sensitive or -resistant EOC, PPC or FTC [56]. The investigators recently reported that trebananib prolonged PFS from a median of 5.4 months in the control arm to 7.2 months (HR 0.66; *p* < 0.001). Trebananib was associated with more AE-related treatment discontinuations and edema events while class-specific anti-VEGF associated AEs were not increased. OS data are still immature [56]. TRINOVA-2 is assessing the combination of PLD with trebananib or placebo in the same setting [57]. TRINOVA-3 is exploring the efficacy of front-line CP plus trebananib or placebo in patients with stage III–IV EOC, PPC or FTC [58].

#### Aflibercept

The novel fusion protein, aflibercept, binds and neutralizes all forms of VEGF-A and VEGF-B and inhibits PlGF activation [59,60]. A phase II multicenter study combined aflibercept with docetaxel every 3 weeks until PD or withdrawal of consent in 46 patients with recurrent ovarian cancer. A high ORR (25 patients, 54%) was achieved, including 10 complete responders, 4 of whom had not recurred at a median of 12 months (range: 5–22 months) post-treatment [52]. Median PFS and OS compared favorably with published data for other investigational agents in this setting (Table 3). The most frequently reported grade 3/4 AEs were absolute neutrophil count (72%), fatigue (50%), dyspnea (22%) and stomatitis (7%) [52].

Single-agent aflibercept has been investigated in two phase II studies in patients with advanced chemotherapy-resistant ovarian cancer and symptomatic malignant ascites. In a single-arm, open-label study in 16 patients, the repeat paracentesis response rate was 62.5% (95% CI: 35.4–84.8) and the median time to repeat paracentesis was 76.0 days (95% CI: 64.0–178.0), with a median PFS of 59.5 days (95% CI: 41.0–83.0) (Table 3) [54]. Two patients experienced grade 3 AEs (hypertension and weight loss in one patient, intestinal perforation in the other patient); no grade 4 events were reported. In a larger randomized, double-blind, placebo-controlled study in 55 patients, aflibercept significantly prolonged the median time to repeat paracentesis compared with placebo (55.1 vs*.* 23.3 days, respectively, 95% CI: 10.6–53.1; *p* = 0.0019) [53], but there was no significant difference in survival between the treatment groups (Table 3). Grade 3/4 treatment-emergent AEs included dyspnea (20% aflibercept vs. 8% placebo), fatigue or asthenia (13% vs. 44%, respectively) and dehydration (10% vs. 12%, respectively). Aflibercept was associated with a higher incidence of fatal GI events than placebo (10% vs*.* 4%, respectively) [53].

#### Nintedanib

The triple angiokinase inhibitor, nintedanib (BIBF 1120), showed a promising PFS benefit in a randomized, placebo-controlled phase II study in 83 women with recurrent ovarian cancer who had responded to chemotherapy, but who were at high risk of further early recurrence (Table 3) [50]. All patients received nintedanib or placebo for 9 cycles or until PD or patient withdrawal. A similar proportion of patients in the nintedanib and placebo arms experienced grade 3/4 AEs (34.9% vs. 27.5%, respectively, *p* = 0.49), but nintedanib-treated patients had significantly more diarrhea, nausea and vomiting (*p* < 0.001 vs. placebo). A significantly higher proportion of nintedanib-treated patients experienced grade 3/4 hepatotoxicity compared with placebo-treated patients (51.2% vs. 7.5%; *p* < 0.001). The randomized, double-blind, phase III AGO-OVAR12 trial investigated the efficacy of front-line nintedanib and CP versus nintedanib and placebo in patients with advanced ovarian cancer [51]. Median PFS was significantly longer in the nintedanib plus CP group (17.3 months) than in the nintedanib plus placebo group (16.6 months) (HR 0.84; 95% CI: 0.72–0.98; *p* = 0.0239). A planned phase II trial will also investigate nintedanib in bevacizumab-resistant, recurrent, or persistent ovarian cancer [61].

#### Cediranib

The oral tyrosine kinase inhibitor, cediranib, which targets VEGF receptor 1, 2 and 3, was active in an open-label phase II trial in 46 patients with platinum-resistant or platinum-sensitive recurrent EOC, PPC or FTC who received treatment until PD, unacceptable toxicity or withdrawal of consent (Table 3) [42]. Due to toxicities observed in the first 11 patients, the dose of cediranib was reduced from 45 mg/day to 30 mg/day. More than 20% of patients experienced grade 3 AEs, including hypertension (46%), fatigue (24%), and diarrhea (13%). Grade 4 AEs were reported in 8.7% of patients. The ICON6 randomized, double-blind, placebo-controlled phase III trial evaluated the addition of cediranib (concurrent or concurrent and maintenance) to platinum-based chemotherapy in women with platinum-sensitive relapsed ovarian cancer. Longer restricted mean PFS was reported in the cediranib concurrent and maintenance arm compared with the placebo arm (11.4 vs. 9.4 months; HR 0.68; *p* = 0.0022) as well as longer restricted mean OS (20.3 vs. 17.6 months; HR 0.70; *p* = 0.049) [43]. The dose of cediranib was further reduced in the ICON6 trial to 20 mg/day following problems with toxicity and compliance at the higher dose. Stage I of the trial will assess safety, while stage II will investigate PFS, OS, toxicity and QoL. Stage II will be conducted after 1 year of follow-up, when approximately 470 patients will have been randomized.

#### Imatinib mesylate

Imatinib mesylate is a tyrosine kinase inhibitor that prevents binding of PDGF to its receptor, PDGFR, and inhibits downstream signaling through Akt [62]. Preliminary efficacy results revealed an ORR of 33% in a phase II study of weekly paclitaxel plus intermittent imatinib in 12 women with recurrent ovarian cancer previously treated with platinum or paclitaxel who had received ≤2 regimens for recurrence [63]. Overall, four grade 3 AEs (diarrhea, edema and two cases of neutropenia) were reported, but there were no grade 4 toxicities.

#### Pazopanib

Pazopanib is a small molecule inhibitor that targets VEGFR, PDGFR and c-kit tyrosine kinases [64]. Data from a randomized, double-blind, phase III trial in 940 women with advanced ovarian cancer who had not progressed after front-line chemotherapy showed a statistically significant PFS benefit for patients receiving pazopanib versus placebo (HR 0.766, 95% CI: 0.64–0.91; *p* = 0.0021) (Table 3) [44]. OS data are not yet mature. Pazopanib treatment was associated with a higher incidence of AEs and serious AEs than placebo (26% vs. 11%, respectively). Three patients receiving pazopanib and one patient receiving placebo experienced fatal serious AEs.

#### Sorafenib

The multikinase inhibitor, sorafenib, has broad activity against tyrosine kinase receptors, including VEGFR and PDGFR, as well as angiogenic factors [65]. Modest activity was shown with sorafenib in an open-label phase II study in 71 women with ovarian cancer or PPC who relapsed within 12 months of platinum-based chemotherapy (Table 3) [48]. Significant grade 3/4 AEs included rash (*n* = 7), hand-foot syndrome (*n* = 9), metabolic disorders (*n* = 10), GI disorders (*n* = 3), cardiovascular disorders (*n* = 2) and pulmonary disorders (*n* = 2). Safety data from this trial preclude further investigation of sorafenib monotherapy in recurrent ovarian cancer. In a randomized, double-blind, phase II trial of maintenance sorafenib in 249 women with EOC or PPC in complete remission after initial platinum-based chemotherapy, PFS did not differ significantly between the sorafenib and placebo arms (Table 3), although there was an imbalance in censoring noted [49]. Dose reductions were more common with sorafenib (67.5%) than placebo (30.1%), and duration of treatment was also shorter in the sorafenib arm (median 17.6 weeks vs. 51.9 weeks with placebo). Similarly, the addition of sorafenib to CP failed to improve 2-year PFS or OS rates in a randomized phase II trial as front-line treatment for stage III/IV ovarian cancer following cytoreductive surgery, but toxicity was increased with the combination regimen [66].

A number of randomized phase II trials are assessing combination regimens with sorafenib in patients with recurrent ovarian cancer. Chekerov and colleagues are assessing the efficacy of topotecan alone, or combined with sorafenib, in platinum-resistant recurrent ovarian cancer. Initial safety results from the first 12 patients suggest that sorafenib plus topotecan is a feasible and safe regimen worthy of continued phase II evaluation [67]. A phase II trial of sorafenib in combination with bevacizumab for advanced ovarian cancer is currently recruiting patients [68].

#### Sunitinib

Sunitinib is another multikinase inhibitor that targets VEGF, PDGF, stem cell factor receptor (KIT) and FMS-like tyrosine kinase-3 (FTL3) [69]. Results of a phase II study demonstrated the activity of single-agent sunitinib in 35 women with recurrent and refractory ovarian cancer (Table 3) [45]. Hypertension and GI symptoms were the most common toxicities. Modest activity was also shown in a phase II trial in 30 patients with recurrent platinum-sensitive ovarian cancer receiving sunitinib intermittently at 50 mg/day for 4 out of 6 weeks [46]. However, the same level of activity was not reported in patients requiring a reduction to 30 mg/day continuous dosing. Fatigue, GI symptoms, hand-foot syndrome and hypertension were the most frequently occurring AEs. More recently, a phase II trial compared continuous and intermittent dosing of sunitinib in 73 women with platinum-resistant ovarian cancer, who had received ≤ 3 prior chemotherapy regimens, and concluded that the intermittent schedule showed greatest activity and should be further evaluated in this setting (Table 3) [47]. The type and incidence of AEs between the treatment groups was similar, and included fatigue, cardiovascular, GI and abdominal symptoms.

## Conclusions

Bevacizumab has demonstrated significant efficacy benefits in four randomized, double-blind, phase III studies in combination with standard chemotherapy for advanced ovarian cancer, both as front-line treatment and in patients with recurrent disease. These findings were confirmed in a meta-analysis of these four studies, which concluded that the addition of bevacizumab to chemotherapy offers meaningful improvement in PFS and ORR in ovarian cancer treatment [70]. The safety profile of bevacizumab was as expected in these studies, most AEs resolved over time, and no new safety signals were reported. Ongoing research has so far failed to identify predictive markers of response to bevacizumab thus precluding the selection of patients most likely to gain benefit. Promising efficacy data have also been published for a number of emerging anti-angiogenic agents including trebananib, nintedanib and pazopanib, which are in phase III development for advanced ovarian cancer. Furthermore, several trials are assessing the efficacy and safety of bevacizumab in combination with novel targeted agents in this setting.

## Competing interests

The authors declare that they have no competing interests.

## Authors’ contributions

GA and DP contributed to the study design, were involved in data interpretation, performed a critical revision of the manuscript and gave their final approval for publication. Both authors read and approved the final manuscript.
